# Biostimulant and Arbuscular Mycorrhizae Application on Four Major Biomass Crops as the Base of Phytomanagement Strategies in Metal-Contaminated Soils

**DOI:** 10.3390/plants13131866

**Published:** 2024-07-05

**Authors:** Pietro Peroni, Qiao Liu, Walter Zegada Lizarazu, Shuai Xue, Zili Yi, Moritz Von Cossel, Rossella Mastroberardino, Eleni G. Papazoglou, Andrea Monti, Yasir Iqbal

**Affiliations:** 1 College of Bioscience and Biotechnology, Hunan Agricultural University, Changsha 410128, China; m15574923792@163.com (Q.L.); xue_shuai@hunau.edu.cn (S.X.); yizili@hunau.net (Z.Y.); 2 Department of Agricultural and Food Sciences, University of Bologna, 40127 Bologna, Italy; pietro.peroni2@unibo.it (P.P.); ross.mastroberardin2@unibo.it (R.M.); a.monti@unibo.it (A.M.); 3 Department of Biobased Resources in the Bioeconomy (340b), Institute of Crop Science, University of Hohenheim, Fruwirthstr 23, 70599 Stuttgart, Germany; 4 Department of Crop Science, Agricultural University of Athens, 11855 Athens, Greece; elpapazo@aua.gr

**Keywords:** humic and fulvic acids, lignocellulosic crop, metal(loid) accumulation, mycorrhizae, phytoextraction, protein hydrolysates

## Abstract

Using contaminated land to grow lignocellulosic crops can deliver biomass and, in the long term, improve soil quality. Biostimulants and microorganisms are nowadays an innovative approach to define appropriate phytomanagement strategies to promote plant growth and metal uptake. This study evaluated biostimulants and mycorrhizae application on biomass production and phytoextraction potential of four lignocellulosic crops grown under two metal-contaminated soils. Two greenhouse pot trials were setup to evaluate two annual species (sorghum, hemp) in Italy and two perennial ones (miscanthus, switchgrass) in China, under mycorrhizae (M), root (B2) and foliar (B1) biostimulants treatments, based on humic substances and protein hydrolysates, respectively, applied both alone and in combination (MB1, MB2). MB2 increased the shoot dry weight (DW) yield in hemp (1.9 times more), sorghum (3.6 times more) and miscanthus (tripled) with additional positive effects on sorghum and miscanthus Zn and Cd accumulation, respectively, but no effects on hemp metal accumulation. No treatment promoted switchgrass shoot DW, but M enhanced Cd and Cr shoot concentrations (+84%, 1.6 times more, respectively) and the phytoextraction efficiency. Root biostimulants and mycorrhizae were demonstrated to be more efficient inputs than foliar biostimulants to enhance plant development and productivity in order to design effective phytomanagement strategies in metal-contaminated soil.

## 1. Introduction

Global climate action reiterates cutting back on greenhouse gas emissions and encourages the transition to a carbon-neutral society, which is why countries around the globe are setting an ambitious timeline to reach net zero emissions. For example, the European Union aims to achieve this goal by 2050, whereas in China target is 2060 [1]. Expansion of the biobased industry both in the EU and China is central to achieving decarbonization targets. However, one primary bottleneck is the sustainable sourcing of feedstock in sufficient quantities with distinct quality traits for specific end-use. Thus, it has been recommended to produce biobased products and advanced biofuels through the cultivation of lignocellulosic crops without compromising the food supply and avoiding any Indirect Land Use Change risks (ILUC) in areas with high soil carbon stocks [2]. The use of contaminated land for growing lignocellulosic crops can therefore offer multiple opportunities: the possibility of sustainable biomass production for the biobased industry and in the long term to progressively remediate the soil [3,4,5,6].

In Europe, about 2.5 million contaminated sites have been estimated, more than half being contaminated with mineral oil and metal(loid)s [7]. Similarly, in the PRC (People‘s Republic of China), almost 16% of Chinese agricultural land is contaminated by metal(loid)s and policies have been formulated to prevent as well as to remediate polluted soils [8].

A range of physical, chemical and biological (e.g., phytoremediation) methods have been tested to reclaim contaminated lands [9]. From the literature, phytoremediation of metal(loid)-contaminated soils was successful at several sites [10,11,12]. However, to date, there are no exclusive plant species for phytoremediating metal(loid)-contaminated soils. In the past, in the EU (i.e., Environment Action Program) as well as in PRC, hyperaccumulators have been extensively studied to extract metal(loid)s and to metabolize/reduce organic xenobiotics and other contaminants [13,14,15,16,17,18,19]. However, the major challenge associated with hyperaccumulators is that they generally grow slowly especially under the limited conditions of contaminated soils, which subsequently leads to poor shoot DW yield and therefore requires a generally long time to reclaim polluted lands [20,21] without any potential income for the farmers, with the exception of some agromining practices such as the phytomining of Ni and its subsequent commercialization as ores [22,23] or the use of Se- hyperaccumulators (especially Brassicaceae) to produce food/feed products biofortified with Se [24,25]. Consequently, resource-efficient industrial multipurpose crops with the ability to grow under challenging conditions are being tested [26] to address the limitations of hyperaccumulator species. This could be performed by ensuring high biomass production, which, even with low-medium element concentrations, can provide high metal(loid) sequestration and can be subsequently processable both for metal recovery and to feed bio-based value chains [27,28]. Among industrial crops, several lignocellulosic species can produce satisfactory yields in contaminated lands with low susceptibility to exposure at medium-high metal(loid) concentrations in soils [29,30]. Moreover, most of these plant species can accumulate a part of the most mobile metals (i.e., Zn, Cd, and Ni) in their shoots (phytoextraction) and immobilize several others (i.e., Cu and Pb) in their root system (phytostabilization) [31,32]. Therefore, today there is considerable interest in the phytomanagement of metal(loid)-contaminated areas cultivating resilient biomass crops, not only to reduce metal(loid) concentrations and labile pool fractions through multiple cropping seasons, but also to provide large amounts of cellulose and hemicellulose for biobased industry and ensuring other ecosystem services such as sequestering soil carbon or preventing water and wind erosion [12,28,29,33]. This strategy is functional in positioning phytomanagement programs as a reliable and viable stand-alone technology for improving soil health and fundamental to implementing sustainable long-term phytoextraction process [34].

Among lignocellulosic perennial and annual biomass crops, miscanthus (*Miscanthus* spp.), switchgrass (*Panicum virgatum* L.), sorghum (*Sorghum bicolor* (L.) Moench, 1794) and hemp *(Cannabis sativa* L.) are characterized by high shoot DW yield, greater resource use efficiency especially water and nitrogen and resilience to biotic and abiotic stresses [6,34,35,36,37,38,39,40]. *Miscanthus* is native to Asia and in China is recognized as an important biomass crop for the local biobased industry with a growth area of over 100,000 ha [41], whereas switchgrass is known as a leading energy grass in North America [42,43,44]. Biomass sorghum (*S. bicolor)* is a C_4_ crop, native to Africa with several subspecies: however, the one with high lignocellulosic and sugar content yield (up to 29 Mg ha^−1^) is considered ideal for biomass production [45,46,47]. Its high productivity and resilience have made it attractive to potentially be grown on metal(loid)-contaminated soils [48]. In fact, it has a discrete accumulation capacity of some metals, especially Zn and Cd [49,50]. Hemp on the other hand is a C_3_ plant native to Asia and has emerged as an important industrial crop both in the EU and China [51,52]. It is one of the lignocellulosic species of greatest interest for phytomanagement as it is known to be able to tolerate high exposure levels to a wide range of metals [36] and produce large quantities of dry biomass (around 12 Mg ha^−1^). This indicates that to step up the soil reclamation efforts, it is essential to carry out field trials with physiologically different crops to efficiently deal with multiple contaminants and pedoclimatic conditions. However, metal(loid)-contaminated soils are often subject to marginal conditions, which can limit the growth potential of even resource-efficient lignocellulosic crop species. Thus, when both biomass production and the phytoextraction of labile metal(loid) pools of the soils are of concern, phytomanagement strategies need to be adjusted to increase the bioavailability and uptake of metal(loid)s while improving the crop performance to make the system economically viable. Application of biostimulants to improve crop growth and enhance the phytoextraction capacity of lignocellulosic crops appears to be encouraging today and can be cost-effective and an environmentally friendly option with a low risk of widespread contamination as in the case of chelating agents [53]. These biological products can stimulate plant nutrition processes independently of the product nutrient content by improving: (i) nutrient use efficiency; (ii) abiotic stress tolerance; (iii) quality traits; and/or (iv) availability of confined nutrients in the soil or rhizosphere (EU Fertilizer Regulation 2019/1009). Furthermore, biostimulants can improve the capacity of plants to deal with complex soil systems, which may comprise multiple contaminants [54]. More importantly, less time and resources are required compared to breeding programs with hyperaccumulator species to achieve valuable results. However, comprehensive information on this crucial aspect is largely lacking both in the EU and China and intensive research work still needs to be performed to optimize strategies for applying these products to the right selected crops and subsequently attempt to achieve tangible positive results under field conditions [53,54]. Currently, biostimulants-oriented phytoremediation research work is largely focused on testing biomass crops on potted soils spiked with contaminants [34,38] where the metal(loid) bioavailability would not be the same as in a real field contaminated soil. Therefore, to understand the real potential of lignocellulosic crops and the use of biological agents for these purposes, it is necessary to conduct trials on real contaminated sites with low to moderate ranges of metal concentrations, the only ones at which efficient phytoextraction is considered feasible [55]. In fact, a wide range of biostimulants are available and it is still not clear what type is appropriate for a certain crop and what type of management is needed to maximize performance in terms of phytoextraction and biomass production under specific contaminated conditions [53]. Moreover, the interactions between contaminants and specific biostimulants alone and in combination to improve the metal(loid) bioavailability, root uptake capacity and subsequent translocation in aboveground biomass need to be more deeply investigated. Indeed, studies evaluating the effects of both single and combined biostimulant application on several crops grown under different soil conditions, are still scarce.

The need to expand sustainable cultivation of lignocellulosic crops on marginal land including metal(loid)-contaminated soils for producing biofuels such as sustainable aircraft fuels and to expand knowledge on which biological products, both applied singly or in combination, could result in a remarkable effect on biomass crop growth and phytoextraction capacity in real contaminated soil make this study pertinent and of great interest. Among the potential biological agents, mycorrhizae (M) were selected in this study because their roles in promoting root system development, metal(loid) and nutrient biding are widely recognized [56,57]. The other two selected categories of biostimulants are: humic and fulvic acid-based root biostimulants (B2) and protein and amino acid-based foliar biostimulants (B1), which are considered among the best-known products for alleviating the potentially deleterious effects of metal(loid)s on plant growth [53]. In particular, humic substances are believed to stimulate crop development through both indirect effects on soil fertility and direct effects on plant metabolism, and have recently been studied on lignocellulosic crops with the potential to increase phytoremediation capacities and limit yield loss. For protein hydrolysates, a positive molecular role in plant primary and secondary metabolism stimulation under stress conditions and in nutrient uptake improvement have been highlighted in an increasing number of studies [54,58]. Thus, this study aimed at evaluating the influence of their applications individually (M, B1, B2) or in combination (MB1, MB2), on the biomass production and phytoextraction potential of four lignocellulosic plant species cultivated in two soils contaminated by metals (Cu, Zn, Cd, Cr, Pb, and Sn) in order to select, for each crop, the treatment that can determine the most tangible effect for subsequent field applications. Two lignocellulosic annuals (*C. sativa* and *S. bicolor*) were tested in Italy and two perennials (*Miscanthus lutarioriparius* and *P. virgatum*) in China, using greenhouse pot experiments and soil collected from the contaminated local fields and primed with the aforementioned biostimulants applied both individually and in combination.

## 2. Results

### 2.1. Biomass Production

**Figure 1:**
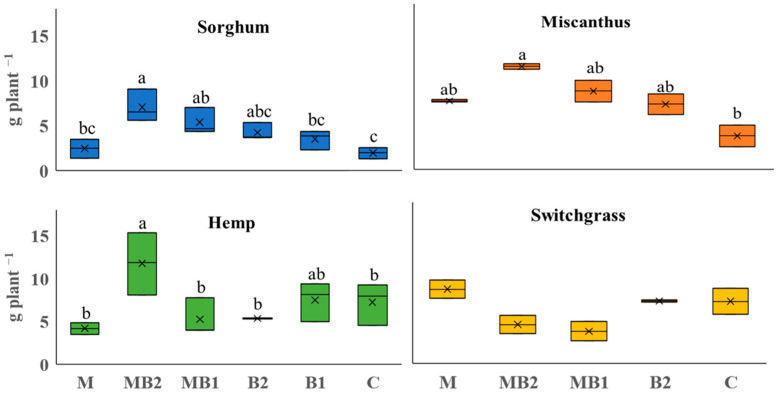
Shoot DW yield harvested at the end of the trials for the annual crops, i.e., sorghum (blue box plots) and hemp (green box plots), and perennial crops, i.e., miscanthus (orange box plots) and switchgrass (yellow box plots). For the annuals, six biological treatments were tested: mycorrhiza (M), mycorrhiza paired with root biostimulants (MB2), mycorrhiza combined with foliar biostimulants (MB1), root biostimulants (B2), foliar biostimulants (B1), and untreated control (C). The same treatments were applied to perennial plants except for foliar biostimulants alone (B1). Within the box plots, x refers to the mean and the horizontal line represents the median. Tukey’s test was used to separate significantly different groups (*p* ≤ 0.05) indicated by the letters above the box plots.

Shoot DW yields varied under-tested treatments for all the crops except switchgrass. For sorghum, hemp and miscanthus, MB2 treatment led to the highest shoot productivity (Figure 1). For sorghum, MB2 treatment doubled the shoot DW yield compared with B1, tripled compared with M and almost quadrupled compared with C. MB1 was also more productive in shoot DW yield than M and C. In hemp, as compared with MB2, the C, B2, MB1 and M treatments produced 38%, 54%, 55%, and 64% less shoot DW yield, respectively. For miscanthus, only the C treatment significantly differed from the MB2 one, delivering one-third of the shoot DW yield of MB2 plants. In switchgrass, no significant differences between treatments were found.

### 2.2. Metal Concentration and Accumulation in Aboveground Biomass

#### 2.2.1. Annuals

**Figure 2:**
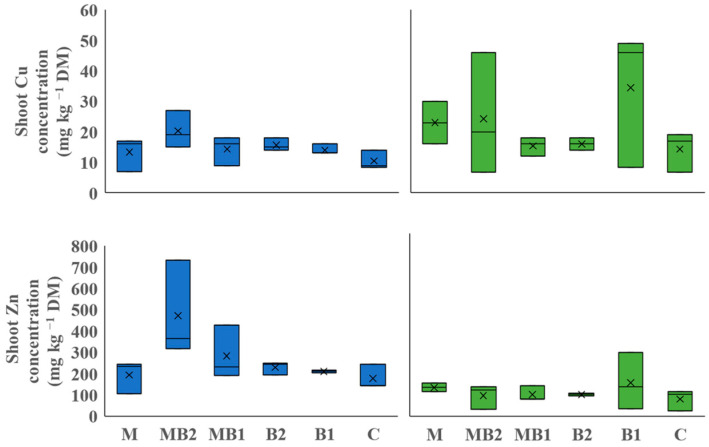
Shoot metal concentration of sorghum (blue box plots) and hemp (green box plots) cultivated in Site 1soil. Mycorrhiza (M), mycorrhiza paired with root biostimulant (MB2), mycorrhiza combined with foliar biostimulant (MB1), root biostimulant (B2), foliar biostimulant (B1), and untreated control (C). Within the box plots, x refers to mean and horizontal line represents median.

**Figure 3:**
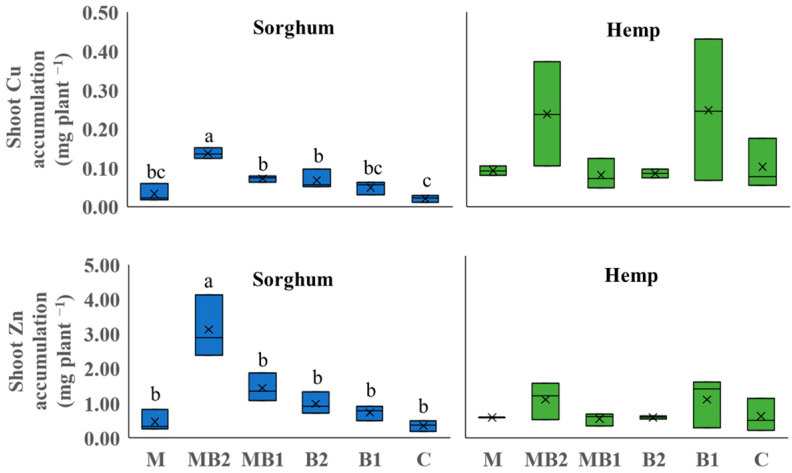
Metal accumulation in the shoots of sorghum (blue box plots) and hemp (green box plots) cultivated in Site 1 soil. Mycorrhiza (M), mycorrhiza paired with root biostimulant (MB2), mycorrhiza combined with foliar biostimulant (MB1), root biostimulant (B2), foliar biostimulant (B1), and untreated control (C). Within the box plots, x refers to mean and horizontal line represents median. Tukey’s test was used to separate significantly different groups (*p* ≤ 0.05) indicated by the letters above the box plots.

For annual plants grown in Italy (Site 1), only Cu and Zn concentrations were above the limit of quantification in shoot digests and quantified in the shoots. However, no significant differences in concentration were observed for both these metals across treatments (Figure 2). The shoot Cu concentrations in sorghum varied from 7 to 27 mg kg^−1^ DM, while for hemp large variation was recorded with values ranging from 7 to 49 mg kg^−1^ DM. For Zn, sorghum and hemp showed even more variation with shoot concentrations ranging from 104 mg kg^−1^ DM to 732 mg kg^−1^ DM and from 24 mg kg^−1^ DM to 278 mg kg^−1^ DM, respectively. Given the total amount phytoextracted in the shoots (Figure 3), MB2 was the most effective treatment in sorghum for both Cu and Zn accumulation. For Cu, the difference in accumulation particularly peaked for MB2 compared with C (6.6 times more for Cu). For Zn, the difference between MB2 and the other treatments was also notable with an increase of 2.2, 3.2, 4.3, 6.6, and 8.9 times more compared to MB1, B2, B1, M and C, respectively. For hemp, no significant differences were found.

#### 2.2.2. Perennials

**Figure 4:**
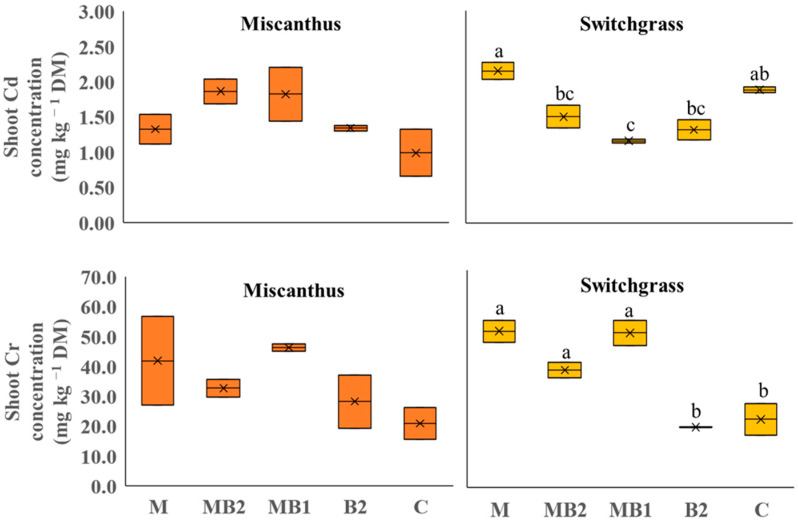
Shoot Cd and Cr concentrations of miscanthus (orange box plots) and switchgrass (yellow box plots) cultivated in Site 2 soil. Mycorrhiza (M), mycorrhiza paired with root biostimulant (MB2), mycorrhiza combined with foliar biostimulant (MB1), root biostimulant (B2), and untreated control (C). Within the box plots, x refers to mean and horizontal line represents median. Tukey’s test was used to separate significantly different groups (*p* ≤ 0.05) indicated by the letters above the box plots.

For perennial crops grown on Site 2 soil, Cd and Cr, were detected and quantified in the aboveground biomass (Figure 4). For miscanthus, no significant differences were found among treatments for both metals. Switchgrass, on the opposite, showed significant differences in metal concentrations.

**Figure 5:**
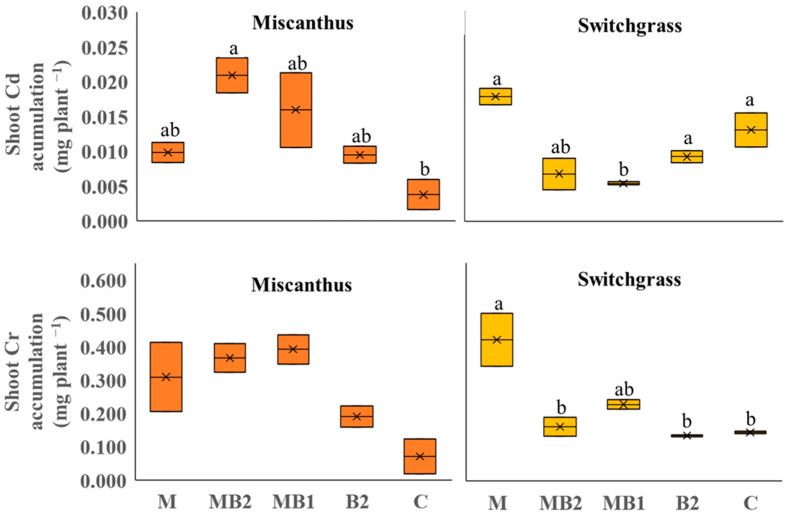
Shoot Cd and Cr accumulation of miscanthus (orange box plots) and switchgrass (yellow box plots) cultivated in Site 2 soil. Mycorrhiza (M), mycorrhiza paired with root biostimulant (MB2), mycorrhiza combined with foliar biostimulant (MB1), root biostimulant (B2), and untreated control (C). Within the box plots, x refers to mean and horizontal line represents median. Tukey’s test was used to separate significantly different groups (*p* ≤ 0.05) indicated by the letters above the box plots.

For Cd, the highest shoot concentration, expressed in mg kg^−1^ DM, was found in M (2.15), which was higher than that of MB2 (1.51), MB1 (1.17) and B2 (1.32), but not significantly different than that of C (1.89). For Cr, all treatments amended with mycorrhiza showed higher shoot Cr concentrations (i.e., M: 50.4, MB2: 37.8 and MB1: 49.8) than others (B2 and C, respectively, 19.5 and 22.0). Given the amount of phytoextracted metals (Figure 5), significant differences were observed for shoot Cd accumulation in miscanthus, which was in line with the results obtained for the shoot DW yield. The MB2 treatment was in fact able to achieve the highest overall accumulation in shoots compared to C (−41%). A similar trend, albeit not significant, was detected for Cr, as the MB2 and C shoots phytoextracted 0.367 mg plant^−1^ and 0.071 mg plant^−1^, respectively. For switchgrass, trends emerged that also seemed in line with what was found for metal concentrations as M treatment was inducing the highest accumulation in shoots for Cd (0.018 mg plant^−1^) and Cr (0.422 mg plant^−1^). For Cd, the difference was significant compared to MB1 (−70%). For Cr, no statistical difference was noted between MB1, unlike MB2 (−61%), C (−66%) and B2 (−32%), where significant differences were recorded.

### 2.3. Phytoremediation Indices

#### 2.3.1. Annual BCF and TF Values

**Figure 6:**
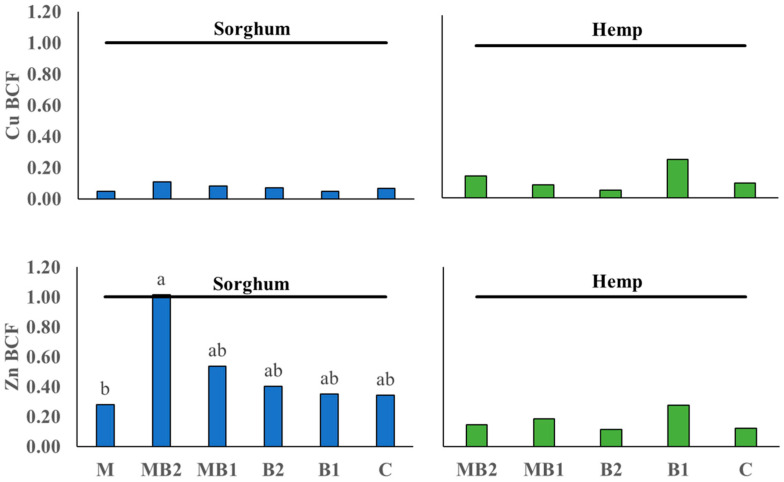
Bioconcentration Factor (BCF) calculated for sorghum (blue histograms) and hemp (green histograms) cultivated in Site 1 soil. Mycorrhiza (M), mycorrhiza paired with root biostimulant (MB2), mycorrhiza combined with foliar biostimulant (MB1), root biostimulant (B2) and foliar biostimulant (B1) and untreated control (C). The black horizontal line identifies the threshold of TF = 1. W Tukey’s test was used to separate significantly different groups (*p* ≤ 0.05) indicated by the letters above the box plots.

The tested treatments for sorghum did not affect the Cu bioconcentration factor (BCF) values, which overall remained well below the threshold of 1 (Figure 6). In contrast, for Zn, a significant difference was noted between MB2 (BCF = 1.01) and M (BCF = 0.28), which was particularly relevant considering that MB2 was the only treatment that allowed sorghum to reach the threshold. In hemp, no significant differences were noted for both metal BCF values and these ones were well below the threshold of 1. In this case, the ranges of both indices showed much more similar values than those noted for sorghum, i.e., in hemp the Cu BCF = 0.05–0.26 and the Zn BCF = 0.11–0.27. The trend was also similar with the highest value recorded for B1 and the lowest recorded in B2, however without statistically significant differences.

**Figure 7:**
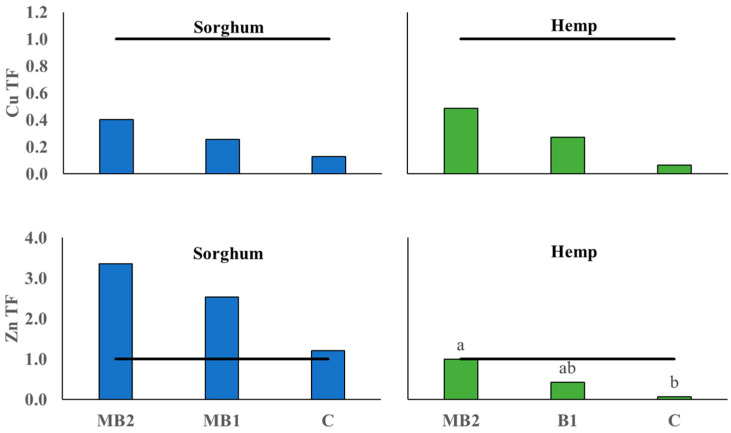
Translocation Factor (TF) calculated for sorghum (blue histograms) and hemp (green histograms) cultivated in Site 1 soil. Mycorrhiza (M), mycorrhiza paired with root biostimulant (MB2), mycorrhiza combined with foliar biostimulant (MB1), root biostimulant (B2), foliar biostimulant (B1), and untreated control (C). The black horizontal line identifies the threshold of TF = 1. Tukey’s test was used to separate significantly different groups (*p* ≤ 0.05) indicated by the letters above the histograms.

Given the translocation factor (TF) values (Figure 7), referring only to both most productive treatments (i.e., MB2 and MB1 for sorghum, and MB2 and B1 for hemp) and the control (C), for both metals in both sorghum and hemp, an apparently increasing trend was found for TFs in relation to shoot DW yield, but only statistically significant differences in hemp for Zn. Here, MB2 practically reached the threshold (TF = 0.99) significantly higher than C, which had a TF value of only 0.07, while B1 was intermediate and did not statistically differ from both MB2 and C treatments, although still below the threshold. For Cu TF values, both sorghum and hemp showed values below the threshold, while for Zn in sorghum, they crossed.

#### 2.3.2. Perennial BCF and TF Values

**Figure 8:**
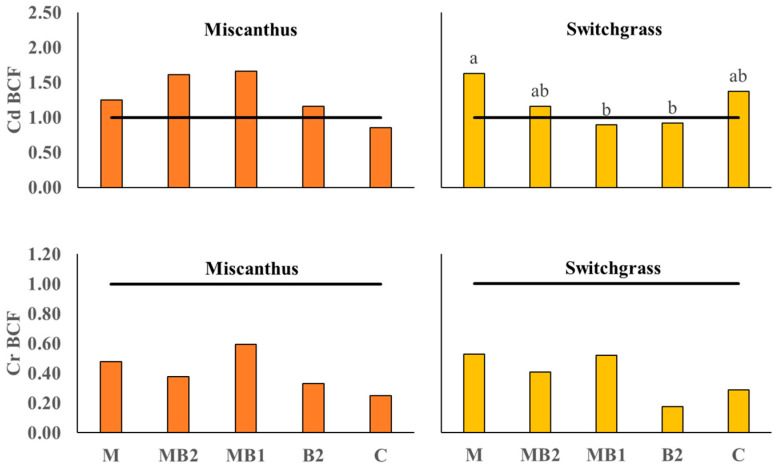
Bioconcentration Factor (BCF) calculated for miscanthus (orange box plots) and switchgrass (yellow box plots) cultivated in Site 2 soil. Mycorrhiza (M), mycorrhiza paired with root biostimulant (MB2), mycorrhiza combined with foliar biostimulant (MB1), root biostimulant (B2), and untreated control (C). The black horizontal line identifies the threshold of BCF = 1. Tukey’s test was used to separate significantly different groups (*p* ≤ 0.05) indicated by the letters above the histograms.

The Bioconcentration Factor for Cd in switchgrass showed significant differences between the applied treatments, but without C being statistically different from any of them (Figure 8).

**Figure 9:**
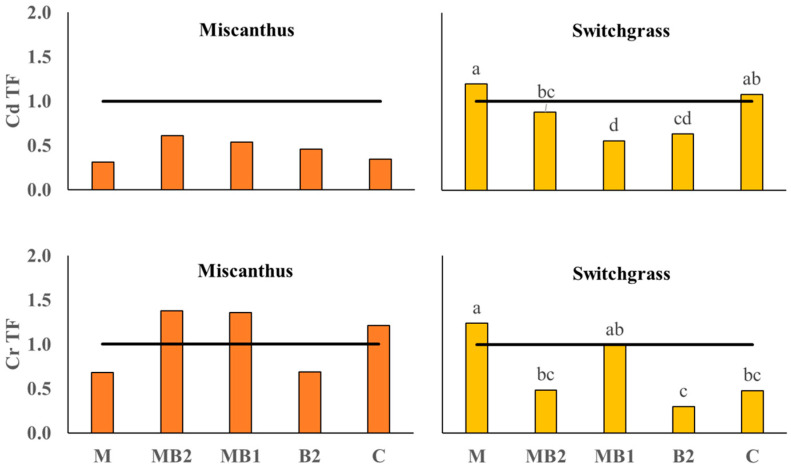
Translocation Factor calculated for miscanthus (orange box plots) and switchgrass (yellow box-plots) cultivated in Site 2 soil. Mycorrhiza (M), mycorrhiza paired with root biostimulant (MB2), mycorrhiza combined with foliar biostimulant (MB1), root biostimulant (B2), and untreated control (C). The black horizontal line identifies the threshold of TF = 1. Tukey’s test was used to separate significantly different groups (*p* ≤ 0.05) indicated by the letters above the histograms.

In this case, M showed the highest index (BCF = 1.63) significantly higher than B2 and MB1, which did not reach the threshold of 1 (respectively, BCF =0.92 and BCF = 0.89). In miscanthus, no significant differences were observed for Cd and all treatments exceeded the threshold of 1 apart from C (BCF = 0.86). For Cr BCF, the treatments did not affect either miscanthus or switchgrass, whose values remained below the threshold. For the TF index of miscanthus (Figure 9), no significant differences emerged between treatments for both metals. In particular, no treatment reached the TF threshold value for Cd, whereas, for Cr, a large variation was recorded with values for MB1, MB2 and C exceeding 1. The TF value was significantly affected by the treatments in switchgrass for both metals: Cd was higher in M (TF = 1.20) compared to all other treatments apart from C (TF = 1.08), which was also higher than MB1 (TF= 0.56). For Cr, there was a positive effect of M (TF = 1.24) in this case comparable only to MB1 (TF = 0.99), while MB2 (TF = 0.49), B2 (TF = 0.30) and C (TF = 0.48) were well below the threshold.

### 2.4. Multivariate Analysis

#### 2.4.1. Sorghum PCA

**Figure 10:**
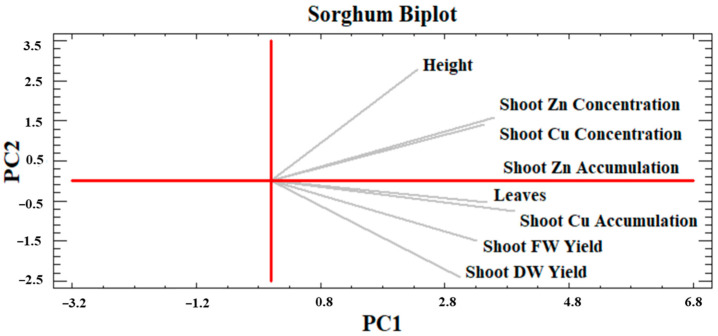
Principal Component Analysis (PCA) of sorghum on relevant plant traits indicated by grey lines. PC1 is the red horizontal axis; PC2 is the red vertical axis. FW and DW refer to fresh and dry weight, respectively.

Two main components, explaining 86.5% of the total variance, were identified (PC1 = 69% and PC2 17.5%) (Figure 10). No variable was negatively correlated with the others as none was placed in an opposite quadrant. This confirmed that plant development and phytoextraction capacity were positively correlated. Furthermore, all the variables were positively driven by the PC1 axis as they were all located on the right quadrants. The shoot Cu and Zn concentrations were partially overlapped in the first quadrant, where they also positively drove the PC2 axis. The shoot height was also found in this quadrant. Both shoot FW and DW yields were placed in the fourth quadrant, as shoot Cu and Zn accumulations and the number of leaves. It is worth noting that the shoot Zn accumulation would exactly correspond to the PC1 axis.

#### 2.4.2. Hemp PCA

**Figure 11:**
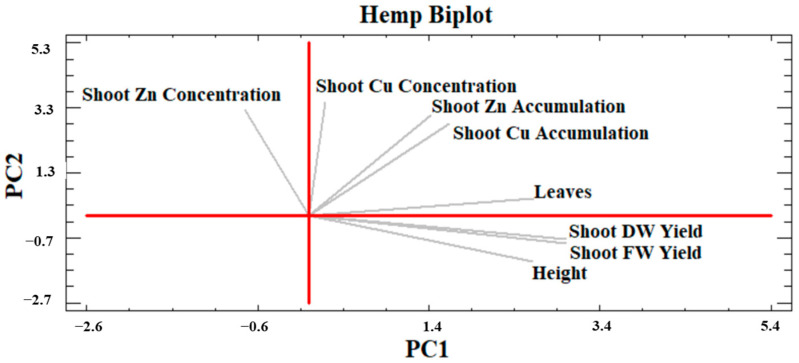
Principal Component Analysis (PCA) of hemp on relevant plant traits indicated by grey lines. PC1 is the red horizontal axis; PC2 is the red vertical axis. FW and DW refer to fresh and dry weight, respectively.

Figure 11 shows the hemp PCA analysis. Two main components explained approximately 88% of the total variance (PC1 = 46.3% and PC2 = 41.7%). The shoot FW and DW yields positively drove PC1 but not PC2, showing a strong correlation with the plant height placed in the fourth quadrant. Moreover, the number of leaves correlated with the shoot FW and DW yields, even though it positively drove PC2. Indeed, this parameter was found in the first quadrant where the shoot Cu and Zn accumulations were also located, closely related to each other. Shoot Zn concentration occurred in the second quadrant in a position almost opposite to shoot FW and DW yields and plant height. Furthermore, shoot Cu accumulation was also located mid-distance from shoot Cu concentration and shoot DW yield, likely due to their use for calculation (same remark for Zn).

#### 2.4.3. Perennial PCA

**Figure 12:**
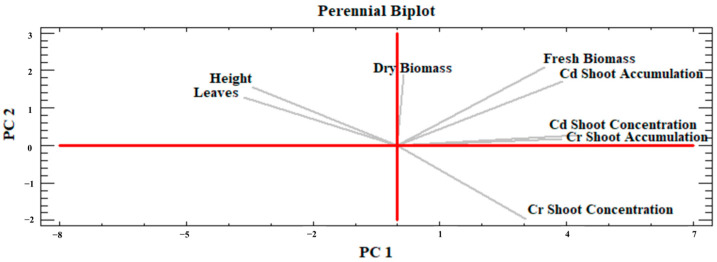
Principal Component Analysis (PCA) of miscanthus and switchgrass on relevant plant traits indicated by grey lines. PC1 is the red horizontal axis; PC2 is the red vertical axis. FW and DW refer to fresh and dry weight, respectively.

For both perennials, the PCA identified three main components capable of explaining 87.7% of the total variance. The first two (Figure 12) however accounted for roughly 72.6% of the variance (PC1 = 54.8% and PC2 = 17.9%). Most variables, especially shoot FW and DW; Cd and Cr shoot accumulation, in addition to shoot Cd concentration, were located in the first quadrant and positively drove both PC1 and PC2. However, plant height and number of leaves were set in the second quadrant showing obvious good correlations with biomass production and positively drove PC2, but negatively drove PC1, and displaying a clear negative correlation with the shoot Cr concentration. The shoot Cr accumulation was clearly crucial as it almost coincided with PC1 and was also closely correlated with shoot Cd concentration, the accumulation of which, however, is also influenced by shoot FW yield.

## 3. Discussion

Phytoextraction is often the most desirable phytoremediation process regarding anthropogenic metal(loid) excess in soils, especially by implementing phytomanagement strategies with high biomass-producing crops, which, being harvested and processed annually, could progressively participate in the effective removal of the bioavailable contaminant fraction and land decontamination with significant economic, social, and environmental advantages if compared to conventional remediation techniques [59,60]. However, this process depends on various soil, climate and crop factors, on which biological agent effects can be multiple [61,62]. The PCA results (Figure 10, Figure 11 and Figure 12) summarized how the investigated crops performed in their above-ground development and phytoextraction capacity when grown in contaminated soil with and without biostimulant applications, alone or in combination. Moreover, the effect of treatments on the metal shoot/soil concentrations was evaluated through the BCF value, while metal translocation efficiency from roots to shoots was evaluated through the TF value (Figure 6, Figure 7, Figure 8 and Figure 9). In fact, the efficiency of a phytoextraction process depends on several pedoclimatic factors and the correct choice of the appropriate crop for the contaminated soil type in combination with the right agronomic inputs. As reported by Vangronsveld et al. [55], the use of biomass crops can be a promising solution under conditions of medium-low soil contamination such as sites 1 and 2, where perennial species can be established for about 15–20 years without local conditions severely compromising biomass yields, which in turn can contribute towards steady phytoextraction of metals over the years. However, some metals such as Cd and Zn are more subjected to the translocation process as they are generally sufficiently available in exchangeable forms in the soil (e.g., free ions, soluble forms), while others such as Cu are less bioavailable (e.g., precipitates with Fe/Mn oxyhydroxides, bound to organic matter and clays), but can be accumulated in belowground biomass [55,63]. In the present study, it was possible to identify the most suitable combinations of lignocellulosic species and biological agents to streamline the design of innovative phytomanagement practices to achieve the highest productivity of valuable biomass and provide durable ecosystem services.

Regarding shoot production, sorghum PCA (Figure 10) affirmed that the number of leaves is a key determinant for the final shoot FW and DW yields because leaves constitute a considerable share of total biomass [64]. Based on the hemp PCA results (Figure 11), shoot FW and DW yields depended mainly on the maximum plant height and to a lesser extent on the number of leaves, as hemp produced smaller leaves. In any case, the MB2 treatment that was the most productive in shoot DW for both plants was also found to have a statistically greater number of leaves (see Table S2 in Supplementary Materials). Moreover, in both hemp and sorghum PCA, the correlation between the number of leaves and Cu and Zn accumulations stands out, as these organs were the main sink for the accumulation of both metals, identified in the literature as essential micronutrients for photosynthesis and numerous enzyme activities (e.g., Cu for superoxide dismutase) [65,66,67,68,69,70,71,72,73,74,75,76,77] and subsequently crucial for crop growth. For sorghum, a synergic behavior of increased Cu and Zn accumulations emerged with plant growth, indicating that the present metal exposure did not limit plant development. Indeed, sorghum, can tolerate shoot Zn concentrations similar to those determined in this study and even higher for Cu [50,68] as its upper critical threshold value in most aboveground plant parts, i.e., 25–30 mg Cu kg^−1^, was not reached [69]. Even for hemp, literature provides evidence that it can tolerate higher metal exposures than those of this study, especially in acidic soils where Zn is much more mobile [67,70]. Hence, in hemp PCA (Figure 11), the almost opposite position of shoot Zn and Cu concentrations with respect to the maximum plant height and shoot DW yield may be due to the dilution effect into the biomass, as in the maximum growth phase Cu and Zn are considerably translocated from roots to new aboveground organs positively determining an effect in increasing shoot DM yield and resulting in greater dilution of the most productive plants [71]. This also explains the trend emerging in hemp TF, especially for Zn (Figure 7) similar to what was observed for the shoot DW yield (Figure 1) and is corroborated by the fact that the Zn concentration in C roots was higher than that found in MB2 and B1 (see Table S3 in Supplementary Materials). However, although the Zn TF for MB2 was > 1, the BCFs of both Cu and Zn were well below the threshold, showing an unsatisfactory phytoextraction behavior of hemp in the tested conditions. However, under alkaline soils as in Site 1, Zn can become a limiting factor due to competition for binding sites. Its restricted absorption and translocation may occur because of reduced Zn^2+^ uptake in favor of other cationic nutrients (e.g., Ca^2+^) [72]. The improved Zn translocation can be crucial to expand hemp-based phytoextraction specifically dealing with Zn-contaminated soils as for Site 1. Under the tested conditions, this result, as well as an increased shoot DW yield, was obtained with the use of MB2 (Figure 1 and Figure 7). However, even the use of protein-hydrolysate (B1) in hemp may reduce pH in the soil pore water and increase Zn solubility and root uptake with subsequent increase in its phytoextraction [70]. In sorghum, the MB2 treatment, capable of maximizing canopy growth, can deliver higher shoot DW yield (Figure 1) along with improved phytoextraction performance (Figure 3). In particular, sorghum would be promising for Zn translocation as all treatments showed a TF > 1 in accordance with the reported literature [68,73], but only through MB2 a BCF > 1 and thus an efficient phytoextraction process, would be achieved (Figure 6). These results provide some new insight into the combined use of some biostimulants, such as MB2 treatment in this study, that increase hemp and sorghum shoot DW yield and streamline Zn translocation which could ultimately expedite the phytoextraction process.

Contrary to sorghum and hemp, perennials were analyzed together in PCA procedure owing to their genetic similarities such as photosynthetic pathway (both are C4 species) and botanical family (Poaceae family). Besides that, the trends recorded in a single PCA also favor this approach. For instance, in miscanthus, treatments had a significant effect on shoot DW yield without greatly influencing shoot metal concentrations and uptakes, except for shoot Cd accumulation, whereas in switchgrass the treatment effect was more pronounced on metal concentrations and accumulations (Figure 1, Figure 5 and Figure 6). In the main parameter cluster found in the first quadrant of the perennial PCA (Figure 12), shoot DW yield would be the leading one driving PC2, showing a stronger correlation with plant height and the number of leaves. These, given the bushy nature of miscanthus and switchgrass, especially in this short-term greenhouse experiment in which stem lignification did not occur [74], are the biometric parameters directly involved in determining the final biomass, compared to the basal diameter and number of stems not considered here. For perennials, treatments significantly influenced plant height, in the case of miscanthus consistent with the higher shoot DW yield of MB2 (Figure 1), and did not significantly affect leaves number, contrary to the findings for annuals (see Table S2 in Supplementary Materials). A PCA-relevant outcome was the opposite trend between such biometric traits and the shoot Cr concentration. Despite the known metabolic and physiological damages that Cr excess can cause in miscanthus and switchgrass, with consequent negative effects on morphological development and crop yield [75,76], no phytotoxicity effects have been seen in the present study. Based on Arduini et al. [77], at progressively higher doses of Cr, even higher than those reported in this study, miscanthus sequestered higher concentrations in the roots than in the shoots, where, in addition, Cr was mainly conveyed towards the older, senescent leaves, while in the younger foliage, this flux was considerably limited. As the senescence stage was not reached by the plants in this study, green leaves accounted for the majority of those counted, explaining the negative correlation between shoot Cr concentration and the crop morphological development also for switchgrass. Furthermore, Cr TFs for miscanthus (Figure 9) were occasionally above or below the threshold without significant differences, thus not indicating a clear phytotoxic condition and without being affected by an apparent clear correlation with the differences detected in root Cr concentrations between MB2 and M (see Table S3 in Supplementary Materials). Differently, in the PCA, Cd was not negatively correlated with plant biometric traits and the correlation between shoot Cd accumulation and shoot FW yield (Figure 12) suggests that Cd would be compartmentalized in younger organs by xylematic flow [78,79]. Miscanthus Cd TF values were below the threshold (Figure 9), showing a well-known excluder behavior, whereby different metals can be sequestered in its roots, sorbed on the iron plaque, or immobilized in the rhizosphere by root exudates produced as an avoidance strategy [80,81,82]. However, in miscanthus Cd BCFs were generally > 1 (with the exception of C) resulting in appreciable phytoextraction efficiency for this metal, contrary to what was observed in Cr (Figure 8). The same conclusions can be extended to switchgrass where, however, M was instrumental in determining efficient Cd phytoextraction with BCF and TF values above the threshold (Figure 8 and Figure 9). These results for Cd phytoremediation indices in M were actually not statistically different from those obtained in C, but the use of M, alone or in combination, resulted in higher root Cd concentrations for switchgrass, in contrast to what was observed in miscanthus where single M showed lower concentrations than humic and fulvic acids, alone or in combination, and than the control (see Table S3 in Supplementary Materials). Indeed, in order to maximize the perennial phytoextraction capacity, the PCA indicated that the choice of the right treatment should have a significant effect on shoot Cr accumulation and Cd concentration, as leading parameters driving the PC1. For switchgrass, M did not affect shoot DW yield, but allowed to maximize shoot Cr accumulation and increase shoot Cd concentration (Figure 4 and Figure 5) resulting in the most promising treatment to facilitate switchgrass-based phytoextraction. For miscanthus, no significant differences emerged for these parameters (Figure 4 and Figure 5). However, Cd accumulation, the third most important variable driving PC1, was highly correlated with the shoot DW yield and these two parameters were maximized by MB2, being the most promising treatment for miscanthus-based phytoextraction, as recorded for sorghum and hemp despite their different genetic and physiological background.

Positive effects of mycorrhization are ascertained for C4 grasses [83], but also for hemp, especially in water stress conditions [84] and high metal exposures [85]. Moreover, evidence of the successful formation of symbioses between the different mycorrhiza and all the plant species considered in the present study has been reported in the literature. [85,86,87,88,89,90]. These beneficial effects on crop growth under expousure to no excessive metalconcentration (Table 1) should also be highlighted in poor soils, especially under low N and P content as detected in our study (Table 2). In fact, to avoid the negative effects of fertilization on symbiosis formation, a fertilizer with low P (5%) was used in our study and only following seeding and inoculum application. However, neither M nor B2 alone significantly affected plant growth, unlike MB2. Ofori-Agyemang et al. [70] showed that at high Cd, Pb and Zn soil concentrations the combined use of mycorrhizae and humic and fulvic acids can reduce the metal bioavailability and their potential toxic effect on miscanthus and hemp roots, improving the shoot yield and shoot metal accumulation. In this study, the positive effect of MB2 in improving metal phytoextraction is more associated with greater plant development and less reduced metal potential phytotoxicity. The positive interactions between both treatments may be multiple. B2 can stimulate root growth and changes in root architecture by stimulating the development of secondary root hairs through auxin-like activity [91,92,93]. This proliferation can create new natural openings and contact sites for spores, promoting mycelium entry into root cells and symbiotic association [94]. Furthermore, the presence of humic substances can stimulate H^+^ ATPase in roots resulting in increased exudation of organic acids, nutrient uptake and development of microbial inoculants [58,92,95]. Finally, humic acids may also participate in increasing P solubilization, thus promoting the role of mycorrhizae [96]. The higher Cd and Cr concentrations in switchgrass with M (Figure 4) are due to the different tolerance and resistance mechanisms developed by mycorrhizal fungi towards metals, including: their adsorption on the cell wall surface through binding with chitin and chitosan; subcellular transport and compartmentalization; and the metal chemical forms transformation through reduction, oxidation and methylation reactions [57]. In particular, the presence of free amino acids and polypeptides with various functional groups on the cell wall confers a negative charge allowing the formation of ionic bonds and the subsequent metal cation chelation [97]. These can then be exported by membrane transporters into the cytosol of plant cells via the fungal hyphal network, which functions as an extension of the host plant’s root system [56]. According to Audet and Charest [98], the application of mycorrhizae in the phytoremediation process can result in an ‘Enhanced Uptake’ at concentrations that are not too toxic to the plant, mainly due to increased nutrient uptake and positive effects on the antioxidant system [57]. Whereas in the case of severe phytotoxicity, mycorrhizae increase their ability to chelate metals in extra-radical hyphae and soil aggregates, implementing avoidance rather than tolerance strategies, which are considerably more metabolically costly. In miscanthus, these effects were not observed and this may be due to the highly specific interaction between plant and fungus and even in very similar species there may be differences due to the functional groups of the host plant, as well as soil fertilization and microbial communities present and interacting with the rhizome [83,99].

## 4. Materials and Methods

### 4.1. Soil Collection and Site Characteristics

Two contaminated sites, one mainly contaminated with Pb, Cu, Ni, Zn, and Sn from illegal dumping of industrial residues in the suburbs of Bologna city (Chiarini; 44°50′ N, 11°28′ E), northern Italy (site-1) and the other one with Cd from the mining activities, but also with a Cr discrete concentration, on the outskirts of Zhuzhou city (27°72′ N, 113°30′ E), southern China (site-2), were investigated. At both sites, contaminants exceeded the local legal threshold limits established for Site 1 by the Italian Legislative Decree 152/06 [100] and for Site 2 by the National Environmental Protection Agency [101] (Table 1).

The soil was taken up at the respective contaminated fields from a depth of nearly 0.8 m considered appropriate to carry out this study with well-known deep-rooted crops [47,102,103,104] before being transported to the greenhouse to setup the corresponding pot trials. Site 1 is characterized by the long-term discharge and deposition of wastes of various origins (i.e., improvised warehouses, small crafts, and processing of raw materials, industrial waste, and residues generated by World War II) therefore not used for any agricultural/recreation activity. Site 2 was formerly a rice-growing area, but is currently abandoned for agricultural activities due to metal contamination, especially Cd, but high concentrations of other elements such as Cr can be detected in the area, caused by the use of polluted irrigation water from smelting activities located nearby.

### 4.2. Soil Preparation and Analysis

At both sites, the same soil preparation procedure was followed for the pot trials as well as laboratory analyses. The fresh soil was passed through a 2 cm sieve to remove the stones and prepare homogenized samples for further soil physicochemical analyses, determination of total metal(loid) contents, and their bioavailable fractions. Soil properties analyses were carried out in accredited external laboratories and are presented in Table 2. Pseudo-total metal(loid) concentrations in soil were determined at both sites by the Inductively Coupled Plasma Optical Emission Spectrometry (ICP-OES) after acid digestion in aqua regia (HCl:HNO_3_, 3:1 *v*/*v*) following the European standard. To determine the bioavailable fraction of metals, Diethylenetriaminepentaacetic acid (DTPA) was used as an extractant at Site 1 following the local authority’s indications [105] and the method detailed by Lindsay and Norvell [106] was adopted. Calcium chloride (0.01M CaCl_2_) was used at Site 2 as considered the most appropriate method for the area. All laboratory analyses were carried out in triplicates.

### 4.3. Biostimulant Treatments

The response of all plant species to three commercial biostimulant products was tested individually and in combination, as presented in Table 3.

### 4.4. Experimental Design and Greenhouse Management

The pot trials were carried out in the greenhouse of the Department of Agricultural and Food Sciences of Bologna University (Italy) for sorghum and hemp with Site 1 soil, and the College of Bioscience and Biotechnology of the Hunan Agricultural University (China) for miscanthus and switchgrass with Site 2 soil. A completely randomized experiment design was used for both trials. Each plant species was planted with three replicates in 12 L pots, with one plant per pot. Periodically, pots were rotated to avoid potential irradiance intensity dilution. Sorghum and hemp seeds were pre-germinated into petri dishes. The substrate used was blotting paper for hemp and sand for sorghum. The Petri dishes were kept in a growth chamber at 20–30 °C for 16 h of light and 8 h of darkness for 5 days before transplanting one seedling per pot. The temperature in the greenhouses was set between 16 and 26 °C with a photoperiod of 12 h of light and 12 h of dark in the first month and 14 h of light and 10 h of dark afterward. The soils were kept constantly at 70–80% of their water holding capacity, weekly measuring the water content in each pot through a Time Domain Reflectometer Sensor (TDR 100, Spectrum Technologies Ltd., Bridgend, UK) in order to reintegrate the estimated water loss with time. M, B1, B2, MB1 and MB2 were tested on sorghum and hemp grown in Site 1 soil, while the same treatments with the exception of B1 (individually applied foliar biostimulant) were tested on miscanthus and switchgrass grown in Site 2, soil. The same application protocol was followed throughout the experiment at each site. In brief, at transplanting time, M was applied at 15 g per pot (corresponding to a minimum number of infectious propagules of 3.000 and a typical average number of infectious propagules of 5.000 according to the Most Probable Number test provided by the product’s technical label), in the M, MB1 and MB2 treatments for each crop species. Once the plants reached a height of 10 cm, B1 was applied as foliar biostimulants in the B1 and MB1treatments, at the first application a dose of 135 mL pot^−1^ was diluted in 0.5 of irrigation water applied to reach 80% of WHC, subsequent doses of 3 mL of product per liter of water to wet the plants fully via sprinklers every ten days. Subsequently, starting from the emergence of the 3rd to 6th true leaves, B2 and MB2 were applied in the irrigation water as root biostimulants. The application frequency was once a week at the rate of 0.5 g pot^−1^ diluted in 0.25 L for the first 4 weeks and 0.7 g diluted in 0.5 L thereafter. During the growth cycle, the foliar treatment was applied a total of 8 times on B1 and MB1, and the root treatment 11 times on B2 and MB2. Cover fertilization was performed at the rate of 3 g pot^−1^ at an NPK ratio of 20-5-10 (Nitrophoska, Eurochem, Zug, Switzerland). The plants were monitored over three months to record plant height and number of leaves periodically. Plants were harvested one week after the 8th application of B1 (foliar biostimulant), and the trial lasted a total of 13 weeks for hemp and miscanthus, whereas 14 weeks for sorghum and switchgrass.

### 4.5. Biomass Collection and Analyses

At the end of the trials, the main biometric plant traits were measured: height (maximum shoot length) and number of leaves. Then aboveground as well as belowground biomass samples were collected for further analyses. For sorghum and hemp grown in Site 1 soil, only the two most productive treatments in dry weight and control were selected for belowground biomass sampling and analysis, whereas for miscanthus and switchgrass grown in Site 2 soil root samples from all treatments were analyzed. Aboveground dry biomass was determined by oven drying at 60 °C until the constant weight was reached. Belowground biomass samples were washed with distilled water before drying as for aboveground biomass. All the samples were milled and then digested with nitric acid (HNO_3_) under pressure. Next, Inductively Coupled Plasma Optical Emission Spectrometry was used to analyze the Pb, Cu, Zn, and Ni total concentrations in sorghum and hemp digestates and Cd and Cr in total concentrations in miscanthus and switchgrass digestates (all values in mg kg^−1^ DM).

For each element, total metal accumulation in shoots was calculated as the product of shoot DW yield (DW) and metal concentration in shoots (MCshoot).

Metal accumulation (mg plant^−1^) = shoot DW (kg plant^−1^) × MCshoot (mg kg^−1^ DM) (1)

The bioconcentration factor (BCF) was calculated as follows to measure the degree of translocation and accumulation of metals from soil to the shoots.

BCF = MC shoot (mg kg^−1^ DM)/MC soil (mg kg^−1^ D.M.) (2)

where: MCshoot is the metal concentration in shoots and MCsoil is the pseudo-total metal concentration in the soil.

Furthermore, the translocation factor (TF) was calculated to measure the degree of translocation of metals from underground tissues to aboveground plant parts.

TF = MCshoot (mg kg^−1^)/MCroot (mg kg^−1^ DM) (3)

For both, BCF and TF, a threshold equal to 1 was identified as a reference value. A BCF ≥ 1 indicates that a plant accumulates in its shoot a metal concentration equal to or higher than that one found in the soil, and may perform well in shoot metal accumulation if its biomass is relevant. A TF ≥ 1 indicates that a plant displays a shoot metal concentration equal to or greater than the root metal concentration, and therefore performs well in shoot translocation of metal. Both indices are considered to evaluate the phytoextraction process [108].

### 4.6. Data Analysis

Influence of the tested treatments on shoot dry and fresh yields (DW and FW, respectively), plant height and number of leaves, metal concentrations in the aboveground and belowground biomass, metal accumulations in the aboveground biomass, bioconcentration factors and translocation factors were subjected to one-way analysis of variance (ANOVA) for each plant species. Before carrying out the analysis, assumptions of normality and homoscedasticity were verified for each parameter, proceeding with appropriate Box-Cox transformations in cases where these assumptions were violated. For parameters where a statistically, significant difference (*p* ≤ 0.05) was noted between treatments, post-hoc Tukey‘s HSD test (*p* ≤ 0.05) was conducted to compare pairs of means (R software, version 4.3.0). Plant fresh yield, height, number of leaves and root metal concentrations are reported in the supplementary materials. After having identified which crop x treatment combinations were more effective in terms of biomass productivity as well as metal accumulation from the soil (BCF > 1) and translocation from the roots to the aboveground tissues (TF > 1), a multivariate statistical analysis was conducted for understanding how different variables influenced each other. The characteristics considered were related to the FW and DW biomass production, biometric traits (height and total number of leaves) and phytoextraction (shoot metal concentrations and accumulations). A principal component analysis (PCA) was, therefore, carried out for these factors, separately for hemp and sorghum whereas, for perennials (miscanthus and switchgrass) combined (Statgraphics Centurion software, version 19).

## 5. Conclusions

The use of root biostimulants such as fulvic humic acids and mycorrhizae can enhance the effectiveness of phytomanagement strategies, increasing shoot DW yield and so indirectly phytoextraction capacity or directly the shoot metal concentrations. These treatments are known to act both directly on soil fertility, indirectly on root growth and plant physiological well-being, as well as in metal chelation and absorption. In fact, these treatments can be particularly useful inputs for the establishment of biomass crops in contaminated and degraded lands, often subject to further marginal conditions.

In this study, an increase in shoot DW yields of sorghum and hemp (annual crops), and miscanthus (perennial) was the key to enhancing the metal phytoextraction process, achieved through MB2, whereas for switchgrass, the treatments tested had no tangible effects on shoot DW yield, but directly influenced shoot metal concentrations and accumulations, maximized through M. Since hemp and sorghum are annual crops, it is essential, for subsequent field applications, to choose treatments that increase shoot DW yield, with a relevant quality according to the biomass processing chains leading to biofuels. At the same time, it would be relevant to maximize metal phytoextraction to progressively improve the soil quality and then its ecosystem services. We found close similar trends between biomass production and translocation efficacy. These trends can be traced back to greater production of leaves for sorghum and hemp, which the related PCAs have underlined as being fundamental organs for Cu and Zn phytoextraction. However, hemp did not exhibit satisfactory phytoextraction efficiency, whereas sorghum exhibited Zn-accumulating behavior, especially through the application of MB2 (BCF, TF > 1). Miscanthus and switchgrass did not show an overall phytoextraction capacity for Cr, although its shoot accumulation was one main PC1 driving parameter in the multivariate analysis. For both species, the results were more satisfactory for Cd accumulation and BCFs.

Experimental studies investigating the additive and synergistic effects of various biostimulants applied alone or in combination are still few, but the application of microbial inoculants with humic and fulvic acids and protein hydrolysates for crop growth and production has been tested consistently. Studies at the field level in these regards are still largely needed to provide clear indications on how to develop phytoremediation techniques while simultaneously producing biomass for the biobased industry. Biological agent selection in pot conditions with real contaminated soil that can result in notable effects on specific crop growth and phytoextraction efficiency is functional for further field experiments in which more tangible effects can be expected.

## Figures and Tables

**Table 1:** Metal(loid) analysis performed on soil samples before the trials. * Indicates that DPTA extraction has been performed, ** indicates that CaCl_2_ has been performed.

Sites	Parameter	Total Content (mg kg^−1^)	Legal Threshold (mg kg^−1^)	Bioavailable Fraction (mg kg^−1^)
Chiarini (Site-1)	Lead (Pb)	159	100	33 *
	Copper (Cu)	137	120	45 *
	Zinc (Zn)	455	150	62 *
	Nickel (Ni)	209	120	9.9 *
	Tin (Sn)	8.8	1	not detected
Zhuzhou (Site-2)	Cadmium (Cd)	1.2	0.3	0.25 **
	Chromium (Cr)	70.4	150	0.01 **

**Table 2:** Soil physicochemical properties on the soil samples from Site 1 and Site 2; (n.d. = not detected).

Parameter	Unit	Site-1	Site-2
Clay	g kg^−1^	149	260
Silt	g kg^−1^	329	340
Sand	g kg^−1^	522	400
pH		8	6
Total limestone	g kg^−1^	160	n.d.
Active limestone	g kg^−1^	52	n.d.
Total organic carbon	g kg^−1^	10	n.d.
Organic Matter	g kg^−1^	17	17
Total nitrogen	g kg^−1^	1	1
Total phosphorus	g kg^−1^	n.d.	2
Total potassium	g kg^−1^	n.d.	4
Assimilable phosphorus	mg kg^−1^	20	n.d.
Exchangeable potassium	mg kg^−1^	318	n.d.
C/N		11	n.d.
EC	µS cm^−1^	n.d.	129
WHC	%	20	25

**Table 3:** Tested treatment abbreviations and compositions.

Abbreviation	Composition
M	Mixture of 7 endo-mycorrhizae * (Symbivit^®^, Symbiom, Letohrad, Czech Republic)
B1	Foliar biostimulant: hydrolyzed peptides (55% *m*/*m*) and amino acids (10% *m*/*m*) in water solution (Siapton^®^, Isagro S.p.A, Milan, Italy)
B2	Powdered water-soluble root biostimulant: humic acids (75% *m*/*m*) and fulvic acids (5% *m*/*m*) (Lonite 80SP^®^, Alba Milagro, Milan, Italy)
MB1	Combination of mycorrhizae and foliar biostimulant
MB2	Combination of mycorrhizae and root biostimulant
C	Untreated control

* M was a mixture of inocula of 7 species of Arbuscular Mycorrhizal Fungi naturally occurring in European soils already used in the past for revegetation and phytomanagement of degraded soils [107]: *Claroideoglomus claroideum*, *Rhizophagus irregularis*, *Claroideoglomus etunicatum*, *Funneliformis mosseae*, *Claroideoglomus lamellosum*, *Septoglomus deserticola* and *Rhizophagus diaphanus.* The supplying company guarantees the absence of pathogens and soil particles in the product. A “mock inoculum” has not been reproduced.

## Data Availability

The raw data supporting the conclusions of this article will be made available by the authors on request.

## Supplementary Materials

The following supporting information can be downloaded at: https://www.mdpi.com/article/10.3390/plants13131866/s1, Table S1. Shoot fresh weight (g plant-1). The table shows the averages for each treatment for each plant. Tuckey’s test was used to separate the significantly different groups (*p* ≤ 0.05) indicated by the letters next to the data; Table S2. Plant Height and Number of Leaves. The table shows the averages for each treatment for each plant. Tuckey’s test was used to separate the significantly different groups (*p* ≤ 0.05) indicated by the letters next to the data; Table S3. Root metal concentrations (mg kg^−1^ DM). The table shows the averages for each treatment for each plant. Tuckey’s test was used to separate the significantly different groups (*p* ≤ 0.05) indicated by the letters next to the data.
