# Supplementary material for: Biostimulant and Arbuscular Mycorrhizae Application on Four Major Biomass Crops as the Base of Phytomanagement Strategies in Metal-Contaminated Soils
Source: Plants (Basel). 2024 Jul 5;13(13):1866. doi: 10.3390/plants13131866 (PMC11244479; doi:10.3390/plants13131866)
Supplement: Supplementary file 1 [file plants-13-01866-s001.zip › plants-3050511-supplementary.pdf]

Supplementary Materials

Table S1. Shoot fresh weight (g plant^{-1}). The table shows the averages for each treatment for each plant. Tuckey's test was used to separate the significantly different groups ($p \leq 0.05$) indicated by the letters next to the data.

Treat	Sorghum	Hemp	Miscanthus	Switchgrass
**M**	11.7 ± 5.1 b	10.0 ± 1.3 b	20.2 ± 1.2 ab	23.2 ± 2.8 a
**MB2**	34.1 ± 7.4 a	32.9 ± 9.6 a	31.7 ± 2.4 a	13.8 ± 3.9 a
**MB1**	26.8 ± 9.8 ab	16.4 ± 6.7 b	25.8 ± 3.7 ab	11.4 ± 5.8 a
**B2**	28.7 ± 9.5 ab	14.1 ± 3.9 b	18.4 ± 4.3 b	20.7 ± 2.0 a
**B1**	22.6 ± 9.0 ab	22.5 ± 8.5 b	*N.A.*	*N.A.*
**C**	12.6 ± 3.4 b	19.1 ± 6.8 b	3.8 ± 3.1 c	18.0 ± 5.4 a

Table S2. Plant Height and Number of Leaves. The table shows the averages for each treatment for each plant. Tuckey's test was used to separate the significantly different groups ($p \leq 0.05$) indicated by the letters next to the data.

Plant	Sorghum		Hemp		Miscanthus		Switchgrass	
**Treat**	**Height (cm)**	**N. Leaves**	**Height (cm)**	**N. Leaves**	**Height (cm)**	**N. Leaves**	**Height (cm)**	**N. Leaves**
**M**	23. 7 ± 3.3 a	9 ± 1 c	56.0 ± 14.1 a	102.0 ± 18.38 b	136.8 ± 7.7 a	17 ± 2.8 a	90.1 ± 8.3b	17 ± 2.8 a
**MB2**	35.5 ± 2.3 a	12 ± 1 a	129.3 ± 34.3 a	213.7 ± 62.7 a	144.1 ± 17.5 a	31.5 ± 16.3 a	101. 8 ± 10.5 ab	31.5 ± 16.3 a
**MB1**	44.8 ± 25.0 a	9.7 ± 0.6 bc	88.7 ± 30.9 a	122.7 ± 24.6 ab	135.0 ± 1.13 a	18 ± 1.4 a	122.7 ± 1.6 a	18 ± 1.4 a
**B2**	32.7 ± 5.7 a	11.7 ± 0.6 ab	76.0 ± 29.7 a	149.5 ± 38.9 ab	142.0 ± 19.1 a	21.5 ± 0.7 a	112.0 ± 3.5 ab	21.5 ± 0.7 a
**B1**	28.7 ± 7.4 a	10 ± 1 abc	104.7 ± 35.23 a	170 ± 23.8 ab	*N.A.*	*N.A.*	*N.A.*	*N.A.*
**C**	24.5 ± 4.6 a	8 ± 0 c	93.7 ± 22.7 a	130 ± 19.3 ab	88.9 ± 36.9 b	11.4 ± 8.0 a	116.1 ± 6.1 ab	11.4 ± 8.0 a

Table S3. Root metal concentrations (mg kg^{-1} DM). The table shows the averages for each treatment for each plant. Tuckey's test was used to separate the significantly different groups ($p \leq 0.05$) indicated by the letters next to the data.

Plant	Sorghum		Hemp		Miscanthus		Switchgrass	
**Treat**	**Cu**	**Zn**	**Cu**	**Zn**	**Cd ***	**Cr ***	**Cd**	**Cr**
**M**	*N.A.*	*N.A.*	*N.A.*	*N.A.*	4.2 ± 0.19 c	61.4 ± 0.4 a	50.4 ± 5.0 a	1.8 ± 0.1 b
**MB2**	50.3 ± 12.2 a	139.3 ± 4.0 a	106.0 ± 134.7 a	208.0 ± 245.8 b	3.6 ± 0.0 a	25.3 ± 10.7 b	37.9 ± 5.6 ab	1.7 ± 0.1 b
**MB1**	80.0 ± 43.1 a	132 ± 66.6 a	*N.A.*	*N.A.*	3.4 ± 0.1 b	34.4 ± 5.2 ab	49.8 ± 5.8 a	2.1 ± 0.1 a
**B2**	*N.A.*	*N.A.*	*N.A.*	*N.A.*	3.0 ± 0.0 a	41.9 ± 3.01 ab	19.5 ± 0.1 b	2.1 ± 0.0 a
**B1**	*N.A.*	*N.A.*	114.0 ± 46.0 a	324.3 ± 168.0 b	*N.A.*	*N.A.*	*N.A.*	*N.A.*
**C**	87.3 ± 43.5 a	147.0 ± 40.6 a	257.0 ± 90.6 a	1018 ± 364.4 a	3.3 ± 0.1 b	41.1 ± 8.8 ab	22.0 ± 7.3 b	1.7 ± 0.0 b
